# Hepatitis E Virus Infection in Sheltered Homeless Persons, France

**DOI:** 10.3201/eid1611.091890

**Published:** 2010-11

**Authors:** Mamadou Kaba, Philippe Brouqui, Hervé Richet, Sekené Badiaga, Pierre Gallian, Didier Raoult, Philippe Colson

**Affiliations:** Author affiliations: Centre Hospitalo–Universitaire Timone, Marseille, France (M. Kaba, P. Colson);; Université de la Méditerranée, Marseille (M. Kaba, P. Brouqui, H. Richet, S. Badiaga, D. Raoult, P. Colson);; Hôpital Nord, Marseille (P. Brouqui, S. Badiaga);; Etablissement Français du Sang Alpes-Méditerranée, Marseille (P. Gallian)

**Keywords:** Hepatitis E virus, homeless persons, HEV transmission, autochthonous hepatitis, injection drug use, France, viruses, dispatch

## Abstract

To determine the prevalence of hepatitis E virus (HEV) infection among sheltered homeless persons in Marseille, France, we retrospectively tested 490 such persons. A total of 11.6% had immunoglobulin (Ig) G and 2.5% had IgM against HEV; 1 person had HEV genotype 3f. Injection drug use was associated with IgG against HEV.

Initially considered a leading cause of acute hepatitis in tropical and subtropical countries, hepatitis E virus (HEV) is endemic to industrialized countries (*1*). Although substantial data indicate that HEV infection is a porcine zoonosis, more information is needed about the epidemiology and transmission of this virus in industrialized countries (*1**–**3*). Homeless persons are at higher risk than other persons for viral hepatitis (A, B, and C) because their lifestyle might include injection drug use (IDU) and poor hygiene (*4*), but data on HEV prevalence among them are scarce (*5**,**6*). In Marseille in southeastern France, ≈1,500 persons are homeless (*4*). Since 2000, shelter-based surveys have been conducted yearly to monitor infectious diseases in homeless persons (*4*). This work determined the prevalence of HEV infection in this population.

## The Study

The surveys were reviewed and approved by the Institutional Review Board (CCPPCRB99/76) (Comité de Protection des Personnes Sud-Méditerranée II; www.cpp-sudmed2.fr/) and the Ethics Committee of the Medical School, University of the Mediterranean, Marseille). Participating homeless persons were examined by a physician and interviewed by using a standardized questionnaire, and serum samples were collected from each participant for laboratory testing. Epidemiologic, clinical, and biologic data that were collected varied from 1 year to another.

Serum samples collected from 490 homeless persons in 2003, 2005, and 2006 in 2 shelters in Marseille (Table A1) were tested retrospectively for immunoglobulin (Ig) G and IgM (EIAgen HEV kits; Adaltis Italia SpA, Rome, Italy) against HEV and for HEV RNA by using an in-house real-time reverse transcription–PCR specific for open reading frame 2 (*7*). HEV RNA sequencing was performed when HEV RNA was detected, and genotype was assigned through phylogenetic analysis of open reading frame 2 partial sequences (*7*). Serologic testing for hepatitis A, B, and C and for HIV were performed by using Axsym Abbott assays (Abbott Diagnostics Division, Wiesbaden, Germany). Statistical analysis was performed by using STATA version 10.1 software (StataCorp, College Station, TX, USA). Pearson χ^2^ test, Fisher exact test, Kruskal-Wallis test, or logistic regression model were used when appropriate.

Mean ± SD age of homeless persons was 43 ± 14 years, and their mean ± SD duration of homelessness was 49 ± 84 months. Most (96.3%) were men and were born in North Africa (40.2%) or in France (33.3%) (Table A1). Previous or ongoing IDU was reported for 4/176 (2.3%). Overall prevalence of anti-HEV IgG and IgM was 11.6% (95% confidence interval [CI] 8.9%–14.8%) (57/490) and 2.5% (95% CI 1.3%–4.2%) (12/490), respectively. Mean optical density ratio (optical density/cutoff value) was 3.0 (range 1.1–6.9) and 2.0 (range 1.1–4.6) for IgG and IgM, respectively. Three (0.6%; 95% CI 0.1%–1.8%) homeless persons were concurrently positive for HEV IgM and IgG, whereas 9 (1.8%; 95% CI 0.8%–3.5%) were positive only for IgM and 54 (11%; 95% CI 8.4%–14.1%) were positive only for IgG.

HEV RNA was detected in 1 homeless person, a 50-year-old man from Romania concurrently seronegative for HEV IgM and IgG and for hepatitis B and C viruses. He reported excessive alcohol intake but no IDU. HEV genotype was 3f (Figure), and sequence analysis showed 98% nt identity with sequences previously recovered from persons in Spain and France. Alanine aminotransferase (ALT) level had been assessed in only 2/12 HEV IgM–positive homeless persons and was elevated in 1 person (177 IU/L), in association with an increased γ-glutamyl transferase level (788 IU/L). Among the 19 homeless persons sampled in 2 different years, 1 seroconverted; he was seronegative for HEV IgM and IgG in 2005 then positive in 2006 (optical density ratio 1.14 and 4.3, respectively). Results of HEV RNA testing were negative in both serum samples, and ALT level had not been tested.

**Figure:**
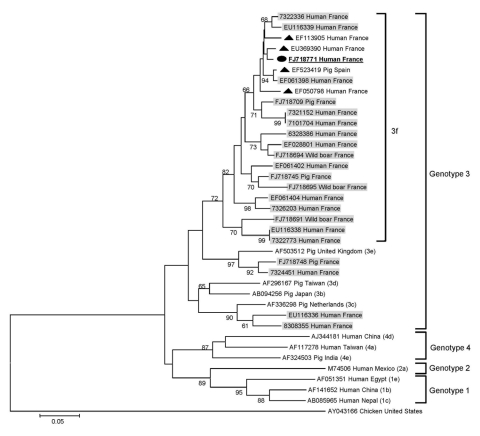
Phylogenetic tree based on partial nucleotide sequences (275 bp) corresponding to the 5′-end open reading frame 2 region of the hepatitis E virus (HEV) genome. Phylogenetic analysis included HEV sequence recovered in the present study (black circle, **boldface** and underlined; GenBank accession no. FJ71877) and sequences corresponding to the HEV sequences hits with the highest BLASTn score (http://blast.ncbi.nlm.nih.gov) to this sequence (black triangles), previously recovered in our laboratory (**boldface**), and of previously determined genotypes and subtypes (*2*) (in parentheses). Shading indicates sequences previously isolated in our laboratory. Bootstrap values >60% of 1,000 resamplings of the data are indicated. Avian HEV sequence AY043166 was used as an outgroup. The names of HEV sequences are labeled as follows: GenBank accession no., host, and country of origin where recovered. Scale bar indicates number of nucleotide substitutions per site.

The prevalence of HEV IgG or IgM in homeless persons did not differ by year of survey; sex; place of birth; or serologic status for hepatitis A, B, or C viruses (Table A1). In addition, mean age, duration of homelessness, and ALT, aspartate aminotransferase, and γ-glutamyl transferase levels did not differ among homeless persons who were positive or negative for HEV antibodies (Table). In the univariate analysis, previous or ongoing IDU (3/4 vs. 19/172; p = 0.006), HIV seropositivity (2/3 vs. 16/183; p = 0.03), and having scabies (6/20 vs. 48/462; p = 0.02) were significantly associated with HEV IgG. In multivariate analysis that used variables that were statistically significant in the univariate analysis as covariates, only IDU was independently associated with anti-HEV IgG (adjusted odds ratio 26.3, 95% CI 2.5–267.1; p = 0.006).

**Table:** Age, duration of homelessness, and liver biochemical test results for 490 homeless persons, Marseilles, France, 2003, 2005, and 2006*

Variable	HEV IgM				HEV IgG		
	Positive	Negative	p value		Positive	Negative	p value
Age, y, mean ± SD	37 ± 12	43 ± 14	0.13		45 ± 15	43 ± 14	0.24
Duration of homelessness, mo, mean ± SD	39 ± 38	49 ± 85	0.36		48 ± 78	49 ± 85	0.28
Liver biochemical tests,† mean ± SD, IU/L							
Alanine aminotransferase levels‡	93 ± 119	32 ± 31	0.99		36 ± 32	32 ± 33	0.49
Aspartate aminotransferase levels§	49 ± 54	34 ± 43	0.99		34 ± 32	34 ± 44	0.79
γ-Glutamyl transferase¶	401 ± 547	65 ± 137	0.92		76 ± 136	68 ± 147	0.44

*HEV, hepatitis E virus; Ig, immunoglobulin. †Assessed only in 2005 for 209 homeless persons. ‡Data missing for 11 persons. §Data missing for 10 persons. ¶Data missing for 9 persons.

## Conclusions

We found that 11.6% (95% CI 8.9%–14.8%) of homeless persons in Marseille were positive for HEV IgG, whereas 2.6% (95% CI 1.3%–4.2%) had HEV IgM or HEV RNA, indicating recent or ongoing HEV infection. The HEV IgG prevalence is similar to that previously found (*6*) in homeless persons in Los Angeles, California, USA (13.5%), and much lower than that found in 98 homeless children in Cochabamba, Bolivia (66.3%) (*5*). This prevalence falls between seroprevalences recently assessed among blood donors in northern (3.2%) and southwestern (16.6%) France (*8**,**9*).

These comparisons should take into account the use of different serologic assays, in addition to differences in epidemiologic settings. Indeed, substantial differences have been observed regarding the performances of some HEV IgG tests (*10*). Moreover, the sensitivity and specificity of the HEV IgG assay used in our study have not been previously evaluated, which warrants a cautious interpretation of the results. A preliminary study conducted in 2008 of 194 blood donors in Marseille with the same assay as that used in the present study found that the prevalence of HEV IgG was 9% (P. Gallian, unpub. data), which is similar to the seroprevalence we found in homeless persons. The HEV IgM assay we used showed good performance in patients with HEV genotype 3 infections in reference to PCR testing (*11*). Thus, sensitivity, specificity, and negative predictive value were 90%, 100%, and 98.8%, respectively. In addition, sensitivities and specificities of this test and of the HEV IgM ELISA 3.0 (MP Diagnostics, Singapore) did not differ significantly.

Although based on a small subset of homeless persons, our finding of the association of IDU with serologic results indicating past HEV infection is intriguing. This result is surprising because the proportion of IDUs in our homeless population was low (2.3%) and much lower than proportions previously reported (9%–83% (*12**–**15*). HEV IgG prevalence of 2.2%–62% has been described in IDUs, but a significant difference with the control group was found in only 2 of these studies (*12**–**15*). HEV IgG prevalence in Sweden was 62% (21/34) in patients who acquired acute hepatitis B through IDU compared with 25% (9/36) in patients with sexually acquired hepatitis B (p<0.005) (*13*). Moreover, in Italy, the prevalence was 5.4% (16/179) in IDUs compared with 2.6% (49/1,889) in the general population (p<0.00001) (*12*). In summary, our data indicate that HEV infection occurs in homeless persons, and further studies are needed to determine whether IDU is responsible for HEV transmission.

## Appendix

**Table A1:** Univariate analysis of associations between variables and positive anti-HEV IgM and IgG testing in homeless persons, Marseilles, France, 2003, 2005, and 2006*

Variable	Total no. persons	IgM testing positive			IgG testing positive	
		No. (%) persons	p value		No. (%) persons	p value
Year of survey						
2003	181	8 (4.4)	0.11		21 (11.6)	0.89
2005	200	2 (1.0)			22 (11.0)	
2006	109	2 (1.8)			14 (12.8)	
Sex						
M	472	10 (2.1)	0.07		55 (11.6)	0.65
F	18	2 (11.1)			2 (11.1)	
Place of birth						
North Africa	197	5 (2.5)	0.98		26 (13.2)	0.94
France	163	5 (3.1)			19 (11.7)	
Eastern Europe	76	2 (2.6)			9 (11.8)	
Sub-Saharan Africa	20	0			2 (10.0)	
Europe (noneastern, except France)	15	0			1 (6.7)	
French overseas departments and territories	12	0			0	
Asia	7	0			0	
History of injection drug use						
Yes	4	0	0.83		3 (75.0)	0.006
No	172	8 (4.7)			19 (11.1)	
History of blood transfusion						
Yes	24	0	1.00		2 (8.3)	0.34
No	158	2 (1.3)			20 (12.7)	
Do not know	17	0			0	
History of surgery						
Yes	103	2 (1.9)	0.27		9 (8.7)	0.20
No	96	0			13 (13.5)	
History of dental care						
Yes	86	1 (1.2)	0.61		8 (9.3)	0.60
No	53	0			5 (9.4)	
Body piercing						
Yes	18	0	0.80		2 (11.1)	0.58
No	154	2 (1.3)			16 (10.4)	
Having tattoos						
Yes	40	1 (2.5)	0.41		2 (5.0)	0.16
No	132	1 (0.8)			16 (12.1)	
History of owning a cat						
Yes	10	0	0.63		1 (10.0)	0.67
No	171	8 (4.7)			20 (11.7)	
History of owning a dog						
Yes	8	1 (12.5)	0.31		0	0.36
No	173	7 (4.1)			21 (12.1)	
History of pruritus						
Yes	117	5 (4.3)	0.38		15 (12.8)	0.39
No	172	5 (2.9)			19 (11.1)	
Having scabies						
Yes	19	1 (5.3)	0.40		6 (31.6)	0.02
No	443	11 (2.5)			48 (10.8)	
History of sexual intercourse with sex workers						
Yes	33	0	0.68		4 (12.1)	0.49
No	154	2 (1.3)			16 (10.4)	
HAV serologic testing results						
Positive	89	2 (2.3)	0.73		11 (12.4)	0.33
Negative	15	0			3 (20.0)	
HBV serologic testing results indicating past or ongoing infection						
Yes	75	2 (2.7)	0.29		9 (12.0)	0.46
No	205	2 (1.0)			22 (10.7)	
HCV serologic testing results						
Positive	24	0	0.52		3 (12.5)	0.52
Negative	437	12 (2.8)			49 (11.2)	
HIV serologic testing results						
Positive	3	0	0.97		2 (66.7)	0.03
Negative	175	2 (1.1)			16 (9.1)	

*HEV, hepatitis E virus; Ig, immunoglobulin; HAV, hepatitis A virus; HBV, hepatitis B virus; HCV, hepatitis C virus.
